# The architecture of functional brain network modulated by driving under train running noise exposure

**DOI:** 10.1371/journal.pone.0306729

**Published:** 2024-08-15

**Authors:** Yashuai Zhao, Yuanchun Huang, Zhigang Liu, Yifan Zhou

**Affiliations:** School of Urban Rail Transportation, Shanghai University of Engineering Science, Shanghai, P.R. China; Institute for Research in Fundamental Sciences, ISLAMIC REPUBLIC OF IRAN

## Abstract

A noisy environment can considerably impact drivers’ attention and fatigue, endangering driving safety. Consequently, this study designed a simulated driving experimental scenario to analyse the effects of noise generated during urban rail transit train operation on drivers’ functional brain networks. The experiment recruited 16 participants, and the simulated driving scenario was conducted at noise levels of 50, 60, 70, and 80 dB. Functional connectivity between all electrode pairs across various frequency bands was evaluated using the weighted phase lag index (WPLI), and a brain network based on this was constructed. Graph theoretic analysis employed network global efficiency, degree, and clustering coefficient as metrics. Significant increases in the WPLI values of theta and alpha frequency bands were observed in high noise environments (70 dB, 80 dB), as well as enhanced brain synchronisation. Furthermore, concerning the topological metrics of brain networks, it was observed that the global efficiency of brain networks in theta and alpha frequency ranges, as well as the node degree and clustering coefficients, experienced substantial growth in high noise environments (70 dB, 80 dB) as opposed to 50 dB and 60 dB. This finding indicates that high-noise environments impact the reorganisation of functional brain networks, leading to a preference for network structures with improved global efficiency. Such findings may improve our understanding of the neural mechanisms of driving under noise exposure, and thus potentially reduce road accidents to some extent.

## Introduction

Safety in urban rail transport has long been a matter of great concern. Research has demonstrated that the primary cause of train accidents is human error [1], with a clear correlation between driver error and train accidents [2, 3]. Urban rail train drivers undertake monotonous driving tasks for extended periods in poorly lit and noisy harsh environments with high workloads, causing fatigue [4]. This negatively impacts their driving performance and, therefore, driving safety. Thus, operators have a significant responsibility for the safety of train operations. Environmental noise has the potential to negatively impact drivers’ physiological health [5] and cognitive and decision-making abilities [6], ultimately threatening operational safety.

Previous research has demonstrated that being exposed to occupational noise can have considerable health effects, with a person’s likelihood of developing high blood pressure and heart disease increasing when subjected to noise levels exceeding 80 dB [7]. As for traffic noise, road noise has an impact not only on the mood and driving behaviour of the driver [8], but can also lead to increased drowsiness [9], and decreased attention and responsiveness [10]. Previous studies on noise have focused on the physiological and psychological effects of noise on drivers [11, 12]. However, there is insufficient understanding of the structure of drivers’ brain networks in response to noise exposure, and few investigations have explored how drivers manage the organization of functional brain networks in high-noise situations.

Electroencephalography (EEG) is a non-invasive measurement of scalp potentials that allows the user’s spontaneous brain activity to be monitored and identified [13]. The cognitive theory perspective states that driving involves a process of perception, judgment, decision-making, and manipulation. The driver’s physical and mental activities are reflected in the EEG signals [14]. Thus, EEG can capture the physiological and psychological alterations of the driver. EEG has been used in numerous studies to assess changes in fatigue while driving. The degree of driver fatigue can be characterised by changes in power in the theta, alpha, and beta bands, as well as by the entropy characteristics of the EEG signal. An increase in human fatigue is typically accompanied by an increase in theta and alpha rhythms and a decrease in beta rhythms [15]. The EEG signal’s sample entropy value typically decreases as fatigue level increases [16].

As researchers have delved deeper into the brain, many studies have shown that the brain is a complex network [17]. Complex networks can adequately account for functional connectivity alterations in the brain [18], as corroborated by research on psychopathologies such as depression [19, 20]. Functional brain connectivity is an important tool for studying driving fatigue in human factors research in the field of transport. Driver performance can be affected by fatigue, which is reflected in the dynamic reorganisation of functional brain networks. Specifically, the temporal global efficiency of dynamic brain networks decreases and temporal local efficiency increases in fatigue [21]. The combination of brain network connectivity features and techniques such as support vector machines, multi-scale convolutional neural networks, and capsule neural networks can achieve high accuracy in recognizing fatigue [22–24]. Therefore, the development of functional brain networks using EEG may enhance our comprehension of the correlation between noise and drivers’ brain topology.

The study employed the weighted phase lag index (WPLI) to evaluate functional connectivity between electrode pairs and construct functional brain networks. We hypothesize that functional brain network configurations in different frequency bands are significantly altered by exposure to train noise during driving. Furthermore, we aim to identify sensitive topological feature indicators of functional brain networks during noise exposure. Compared to previous studies on the impact of noise on driving safety, this paper offers a distinctive contribution in the following three aspects. (1) We investigate whether driving under noise exposure results in changes to brain network properties. To achieve this, we explore topological shifts in brain networks under various noise levels using complex networks. (2) We analysed phase synchronisation using WPLI, a metric that is more robust to volume conduction effects. (3) Graph-theoretic analysis was employed to enhance comprehension and intuition of the regulatory reorganization of brain networks whilst driving under noise exposure.

## Materials and methods

### Participants

To minimize inter-subject variation, a cohort of 16 participants was recruited for this study, consisting of both undergraduate and graduate students aged between 21–25 years. As train drivers are mostly male, the subjects recruited for this study were all male. All subjects self-reported as being in a good state of health, with normal or corrected vision, normal hearing, and no major medical history. Participants were instructed to abstain from consuming alcohol, caffeine, and drugs for 24 hours prior to the experiment and to get a minimum of 8 hours of sleep the night before.

This study was approved by the Ethics Committee of the Shanghai University of Engineering Science under the approval number: EST-2023-022. All participants were adults and all completed a written informed consent form. Subjects were recruited from 15 March 2023 to 15 April 2023.

### Task and procedure

**Fig 1:**
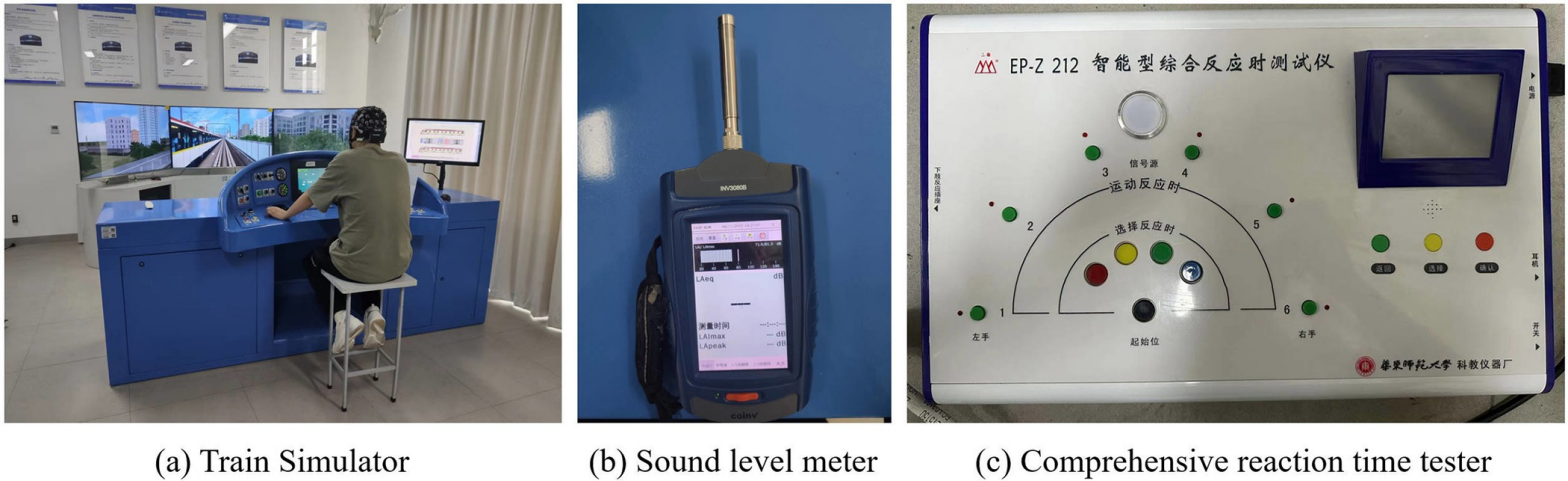
Experimental equipment.

As shown in Fig 1, this study used a rail transit driving simulation system as the experimental platform to play the field-collected underground operation noise through loudspeakers. The experiment was conducted in an experimental environment with controlled temperature and no echo in the house. A handheld sound level meter model INV3080B-SLM from COINV was used to measure the noise exposure level at the subject’s ear. The comprehensive reaction time tester developed by East China Normal University was used to measure the subjects’ choice reaction time. Measuring reaction time involves the subject placing their finger on the button in the starting position. They must then quickly press the button corresponding to the randomly lit colour of the signal source. The reaction time is calculated as the time difference between releasing the start button and pressing the corresponding colour button.

**Fig 2:**
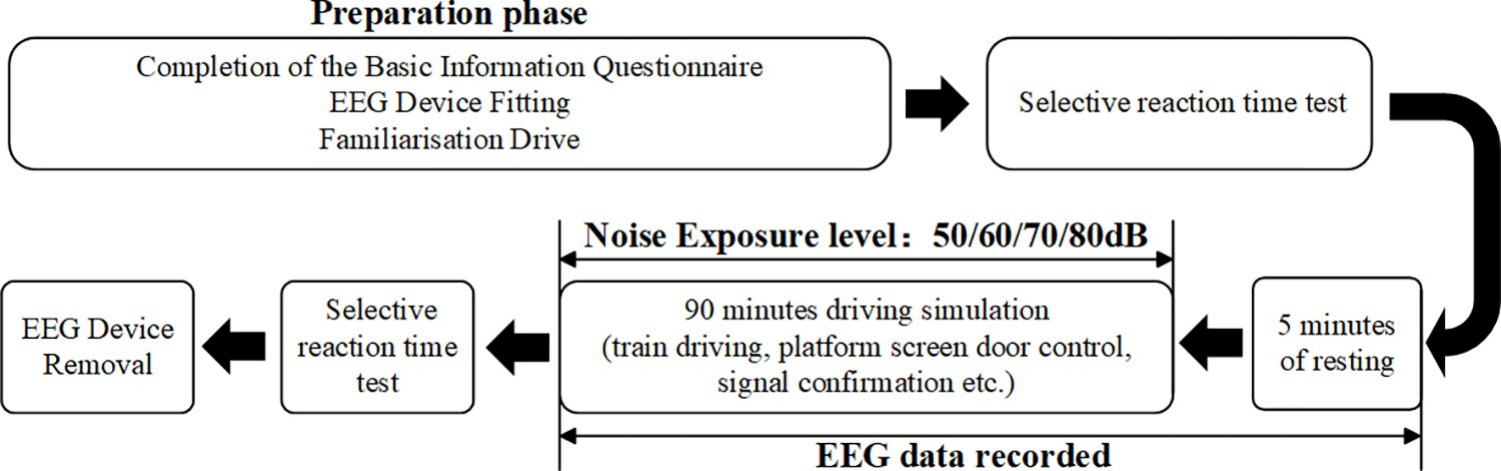
Overview of experimental design.

To eliminate the influence of biological rhythms, the experiment will take place at a uniform time, 14:00 to 16:00 daily. The driving simulation experiment will expose participants to four distinct noise levels (50/60/70/80 dB) along a predetermined line of the Shanghai Railway, simulating both elevated and underground sections under sunny weather conditions. During driving, the operators must control the acceleration and deceleration of the train to ensure it runs smoothly without exceeding the speed limit of the track section. Upon arriving at the station, it is imperative to confirm signals and manually stop the train before loading and unloading passengers. Fig 2 shows the whole test procedure. Before the commencement of the experiment, participants are required to complete a basic information questionnaire and an experimental informed consent form. They will also be informed about the experimental procedure. Following this, participants will wear an EEG cap and complete a 20-minute driving simulation exercise to reduce any familiarity differences with the driving simulator. After commencing the experiment, the participants first took a reaction time measurement, then had a 5-minute rest period, and then completed a 90-minute simulated driving task in a noise-exposed situation, with reaction time measurements taken again at the end of the driving task. The EEG signals were recorded for a duration of 95 minutes, from the start of the resting period until the end of the driving period. The noise was only played back during the driving period. Moreover, individuals were restricted from consuming food and beverages, using mobile devices or conversing with the experimenter during the driving simulation. Upon concluding the simulation, the subjects were compensated with a specific sum of money.

### Data acquisition and processing

**Fig 3:**
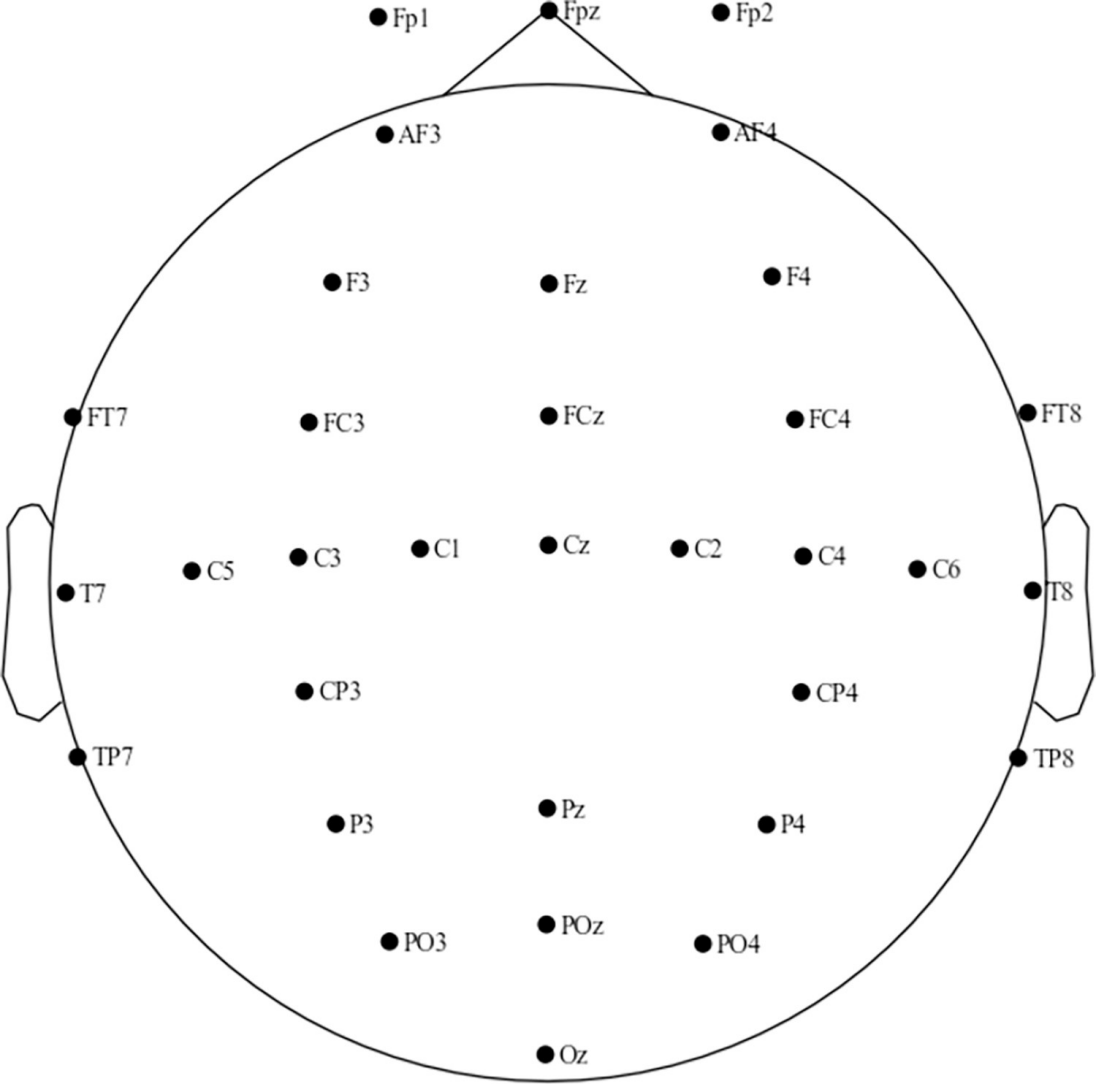
Locations of EEG electrodes.

Throughout the study, EEG signals were recorded using 33 channels on the NeuSen W-series wireless EEG cap from Neuracle. As illustrated in Fig 3, 33 EEG electrodes are positioned at Fpz(1), Fp1(2), Fp2(3), AF3(4), AF4(5), Fz(6), F3(7), F4(8), FCz(9), FC3(10), FT7(12), FT8(13), Cz(14), C1(15), Cz(14), C1(15), C2(16), C3), FC4(11), FT7(12), FT8(13), Cz(14),C1(15), C2(16), C3(17), C4(18), C5(19), C6(20), T7(21), T8(22), CP3(23), CP4(24), TP7(25), TP8(26), Pz(27), P3(28), P4(28), P3(29), P4(29), P4(31), P4(31), P4(31), P4(31), P4(31), P3(32), P4(33), P4(35), P4(36), Pz(37), P3(38), and P4(39) (28), POz (30), PO3 (31), PO4 (32), and Oz (33), according to the standard 10–20 system. Electrode impedance was kept below 15 KΩ. We preprocessed the recorded EEG signals in this investigation utilizing MNE-Python [25]. The EEG signals obtained were initially re-referenced with an averaging reference method. Subsequently, a high and low pass filter was applied to preserve the EEG signals ranging from 1 to 30 Hz. This was carried out to facilitate the construction of functional brain networks. The original sampling rate of NeuSen W was 1000 Hz. However, it was adjusted to 256 Hz in this study to hasten data processing. To reduce the influence of non-EEG factors on EEG signals, this study employed the Independent Component Analysis (ICA) algorithm for examining artifactual components, such as eye movements and blinks, and disposing of them using the ICLabel model [26]. The filtered EEG data was then separated into three frequency bands—theta (4–8 Hz), alpha (8–13 Hz), and beta (13–30 Hz)—by applying bandpass filtering [27].

### Functional connectivity analysis

Functional connectivity assesses the statistical dependence of physiological time series that are recorded across different regions of the brain. It enables analysis of the functional relationships between brain regions. Among these, phase synchronisation indices are frequently employed to assess brain functional connectivity. Phase locking value (PLV), phase lag index (PLI), and weighted phase lag index (WPLI) are common phase synchronisation indices [28]. Among these methods, the PLV is capable of detecting the degree and directionality of phase coupling, but it does not address the issue of volume-conduction during EEG acquisition. The PLI demonstrates better resistance to volume conduction issues. Nevertheless, it is more susceptible to signal amplitude disturbances. In contrast, the WPLI is not only insensitive to volume conduction and noise, but it also performs better with smaller sample sizes [29]. Therefore, the study utilised the weighted phase lag index (WPLI) to measure the connectivity of functional brain networks. Assuming that X is the cross-spectrum between x(t) and y(t), the WPLI is defined as:

$$\mathrm{WPLI}=\frac{\left|E\{ℑ(X)\}\right|}{E\{|ℑ(X)|\}}=\frac{\left|E\{|ℑ(X)|\mathrm{sgn}(ℑ\{X\})\}\right|}{E\{|ℑ(X)|\}}$$ (1)

Where ℑ(*X*) is the imaginary part of the cross-spectrum between x(t) and y(t). 0≤WPLI≤1, 0 indicates a lack of phase synchronisation between the two signals and 1 indicates complete phase synchronisation.

**Fig 4:**
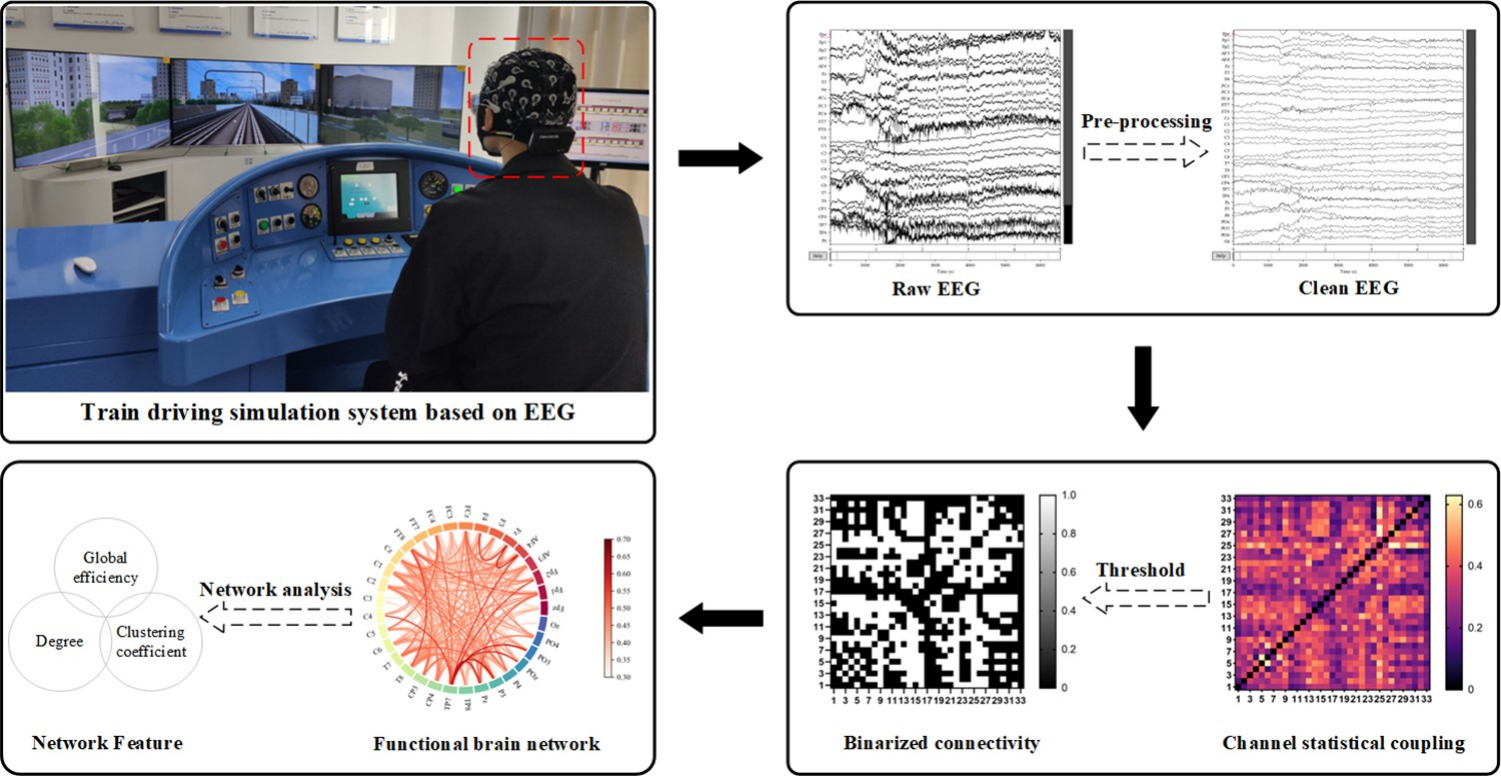
The construction process of functional brain network.

As illustrated in Fig 4, the brain network construction process, in this study, the EEG signals at the end of simulated driving for all subjects were extracted for pre-processing, and the WPLI values between electrodes under θ, α, and β bands were calculated separately to form the WPLI matrix. The WPLI matrices under the same background noise will be overlaid and averaged to obtain the connectivity matrix for that specific noise condition. To minimize false links and insignificant weak associations resulting from interference, an appropriate threshold must be established to transform the connectivity matrix into a binary matrix [30]. The brain network can subsequently be constructed using this binary matrix. WPLI values below the specified threshold are set to 0, indicating no connectivity between the two nodes. Conversely, when WPLI values exceed the threshold, they are set to 1, indicating the presence of connectivity between the two nodes. In this study, each electrode is defined as a node. In this paper, we have employed the threshold strategy to ensure connectivity of the network while keeping network efficiency and cost in mind. As per our selection criteria, the network density S is set at less than 0.5 [31], and the average degree K is greater than or equal to ln(N) ≥ 0 (where N represents the total number of nodes). Additionally, the connectivity matrix has been binarised using varying thresholds for different frequency bands. The McNemar test is frequently employed in medical research to ascertain disparities in binary samples [32]. In this investigation, the McNemar test was utilised to assess variations in brain network patterns across diverse noise conditions in each frequency band, using SPSS on the binarised matrix following thresholding.

### Complex network and graph analysis

Professor Stam and colleagues from the Netherlands [33, 34] used MEG to analyse the properties of brain networks in healthy individuals and EEG to study the functional brain networks of Alzheimer’s patients. Their research confirmed that the functional networks in the human brain are complex, with a "small world" character. Consequently, using graph theory to analyse these complex networks is an effective way to investigate functional brain networks [35].

Global efficiency was selected as a metric to quantify the overall characteristics of functional brain networks [36]. Frequently, global features are expressed in terms of feature path lengths; nevertheless, disconnected points in thresholded brain networks can affect the computation of feature path lengths. Therefore, global efficiency was chosen to assess the capability of functional brain networks to transmit and process information. Global efficiency (E) is defined as:

$$E=\frac{1}{N(N-1)}\sum_{i\neq j}\frac{1}{L_{ij}}$$ (2)

Where N represents the total number of nodes within the network, and *L*_*ij*_ denotes the shortest path length between nodes i and j. Increased global efficiency indicates enhanced transmission and processing capability of the network.

Node degree and clustering coefficients were selected to quantify local features of functional brain networks [37]. Degree is a fundamental metric in complex network theory and represents the number of edges connected to a particular vertex, that is to say, the number of edges passing through that vertex, the degree(K) is defined as:

$$K_{i}=\sum_{j=1}^{N}h_{ij}$$ (3)

Where *h*_*ij*_ is the element of the binary matrix created, N represents the total number of nodes in the network. *h*_*ij*_ is assigned 0 to represent disconnected nodes i and j, and assigned 1 for connected nodes i and j. The significance of a node’s degree value correlates with its importance within the network.

The clustering coefficient is utilised in complex networks to quantify the level of node aggregation. Specifically, the clustering coefficient of a given node i, is expressed as the ratio of the actual number of edges connecting that node to its neighbours to the maximum number of possible connections between them. The clustering coefficient (C) is defined as:

$$C_{i}=\frac{{2e}_{i}}{k_{i}(k_{i}-1)}$$ (4)

Where *k*_*i*_ represents the number of neighbouring nodes of node i, and *e*_*i*_ represents the number of actual connected edges with node i among its neighbouring nodes. A higher clustering coefficient indicates that the respective node is more significant in the network.

Graph-theoretic feature metrics of complex networks were calculated using the package *NetworkX* [38] for this study. The samples did not meet normality standards after the Shapiro-Wilk test and as a result, the Wilcoxon signed-rank test was employed in this paper to examine local differences in feature metrics across various noise conditions within each frequency band.

## Results

### Functional brain networks

**Fig 5:**
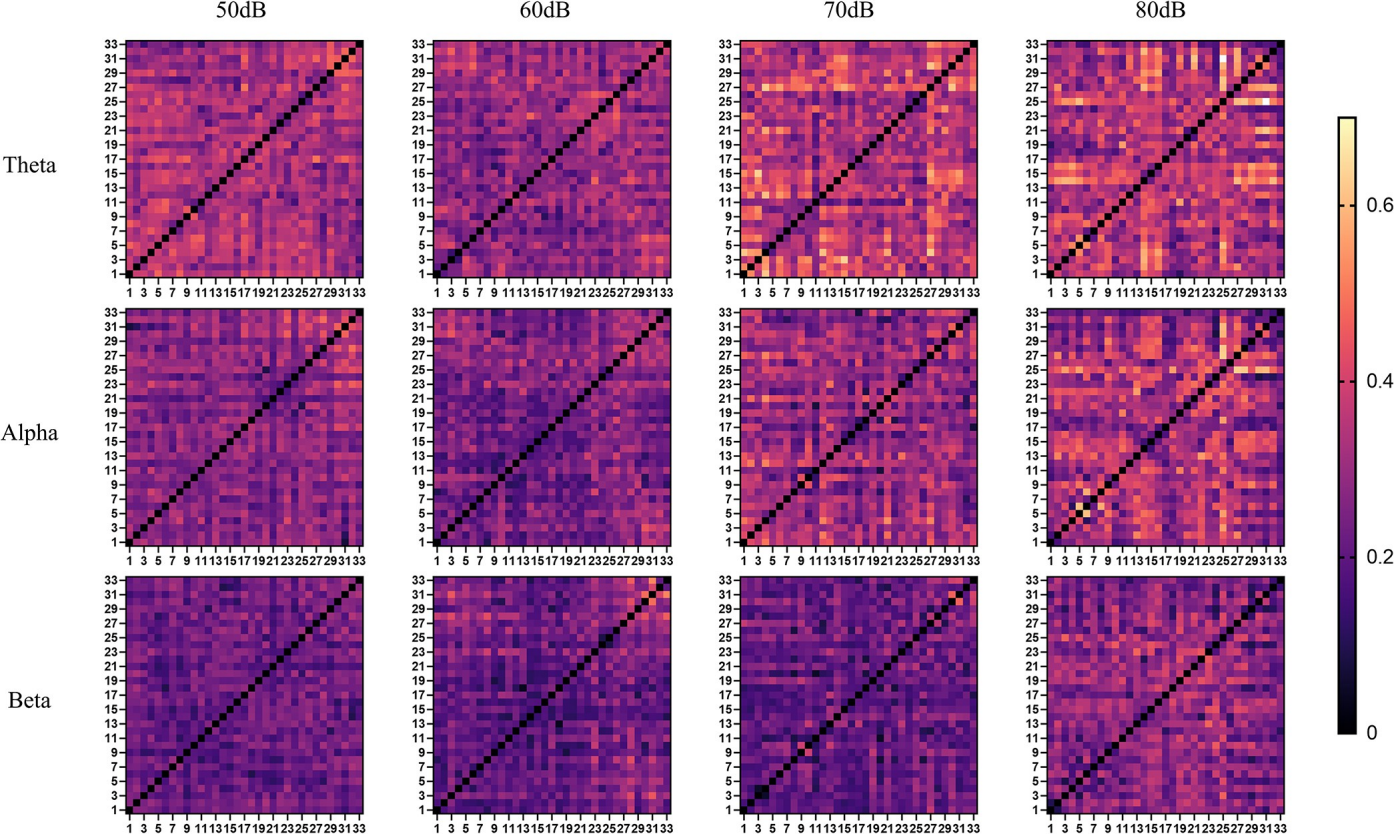
WPLI neighbourhood matrix at different noise levels. The numbers 1–33 in the figure represent the 33 electrodes mentioned above. Each block of colour represents the WPLI value between each pair of electrodes. The more yellow the colour, the greater the WPLI value.

Fig 5 shows the mean adjacency matrix calculated using the WPLI method at different noise levels (50, 60, 70 and 80 dB). These matrices exhibit the frequency bands of theta, alpha, and beta, respectively, where the electrode numbers are allocated on the horizontal and vertical axes. The more yellow the colour, the greater the WPLI value, indicating amplified phase synchronization. Examination of Fig 5 reveals variations in the adjacency matrix associated with different noise levels. In particular, the theta and alpha bands show significantly elevated WPLI values at 70 and 80 dB, in contrast to 50 and 60 dB. This suggests that higher WPLI values in high-noise situations indicate high levels of phase synchronisation.

**Fig 6:**
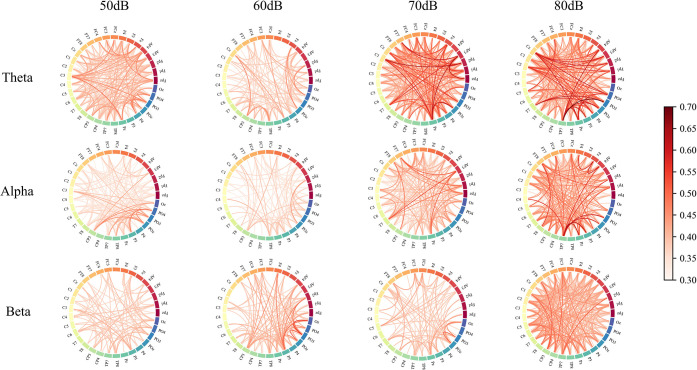
Functional brain network topology at different noise levels. The line segments in the figure represent the presence of connections between the areas represented by the electrodes. The darker the colour, the stronger the connection.

In this study, we transformed the adjacency matrix above to a binary matrix by applying a thresholding technique before plotting the topology of the functional brain network. Fig 6 excludes the weak connection between two electrode pairs with WPLI values below the threshold in Fig 5, and the connectivity between nodes (electrodes) can be clearly seen based on the connection density of the network. The strength of the connectivity between two nodes in the brain network is indicated by the darker the colour. Fig 6 illustrates that increased noise levels result in heightened brain network connectivity and strength, particularly noticeable in the theta and alpha frequency bands. This results in a significant increase in the density and strength of the brain network. The effect of noise on brain network connectivity and strength is markedly more pronounced at noise levels greater than 70 dB for both the theta and alpha frequency bands. We conducted a noise level comparison at 50 dB and 60 dB, and our findings suggest that brain networks exhibited marginally stronger connections at 50 dB. However, for the beta band, the impact of noise was found to be more pronounced at 80 dB.

To confirm the noted variances, this study analysed brain network distinctions in every frequency band under diverse noise conditions using the McNemar test on the thresholded binary matrix. Table 1 presents the results of the McNemar test. Except for those under the beta frequency band, where there was no significant difference between the comparisons of 50 dB with 60 dB and 70 dB, the comparison of noise levels demonstrated significant differences. This indicates that different types of noise have an impact on the brain networks. The findings align with the changes in brain network topology depicted in Fig 6.

**Table 1:** The results of the McNemar test.

Frequency band	Comparison	McNemar test (p-value)
Theta	50 dB vs 60 dB	**<0.0001**
	50 dB vs 70 dB	**0.0011**
	50 dB vs 80 dB	**0.0035**
	60 dB vs 70 dB	**<0.0001**
	60 dB vs 80 dB	**<0.0001**
	70 dB vs 80 dB	0.5907
Alpha	50 dB vs 60 dB	**<0.0001**
	50 dB vs 70 dB	**<0.0001**
	50 dB vs 80 dB	**<0.0001**
	60 dB vs 70 dB	**<0.0001**
	60 dB vs 80 dB	**<0.0001**
	70 dB vs 80 dB	**0.0001**
Beta	50 dB vs 60 dB	0.0589
	50 dB vs 70 dB	0.9317
	50 dB vs 80 dB	**<0.0001**
	60 dB vs 70 dB	0.0565
	60 dB vs 80 dB	**<0.0001**
	70 dB vs 80 dB	**<0.0001**

Bold text represents significant results (p < 0.05).

### Graph-theoretic characterisation

**Fig 7:**
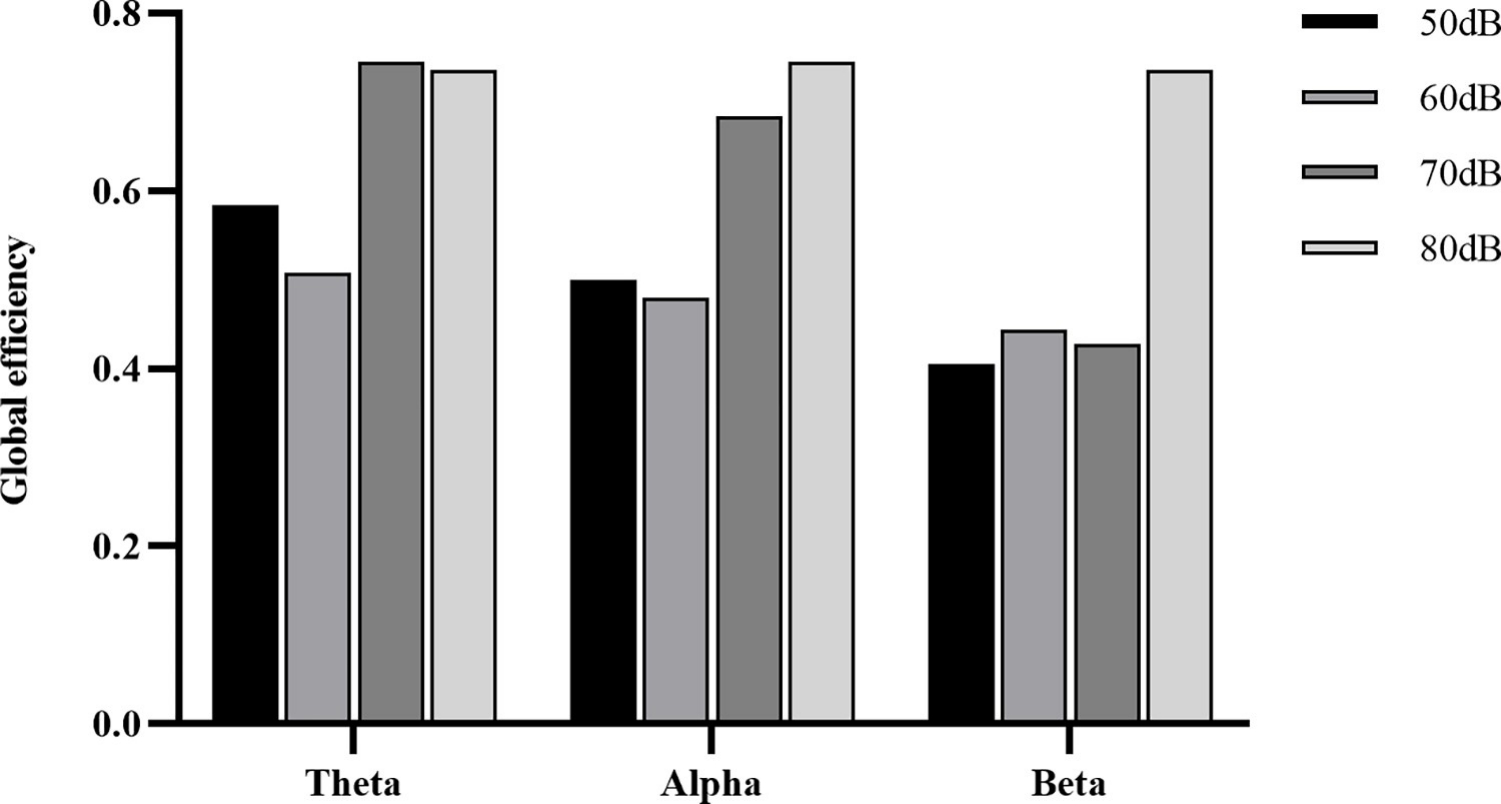
The global efficiency metrics of functional brain network at different noise levels.

Functional brain networks, as a type of complex networks, can be characterised in terms of the statistical properties of complex networks to describe the changes in brain networks. In this study, the graph-theoretic characterisation of brain networks in each frequency band under different noise levels was performed and the global efficiency metric was calculated, and the results are shown in Fig 7. For the theta and alpha frequency bands, the global efficiency of the brain network in 70 dB and 80 dB noise environments is the highest. While for the beta frequency band, it is the brain network under an 80 dB noise environment that has a higher global efficiency than the former. It can also be seen that the global efficiency of the network in a 50 dB noise environment is slightly higher than 60 dB.

**Fig 8:**
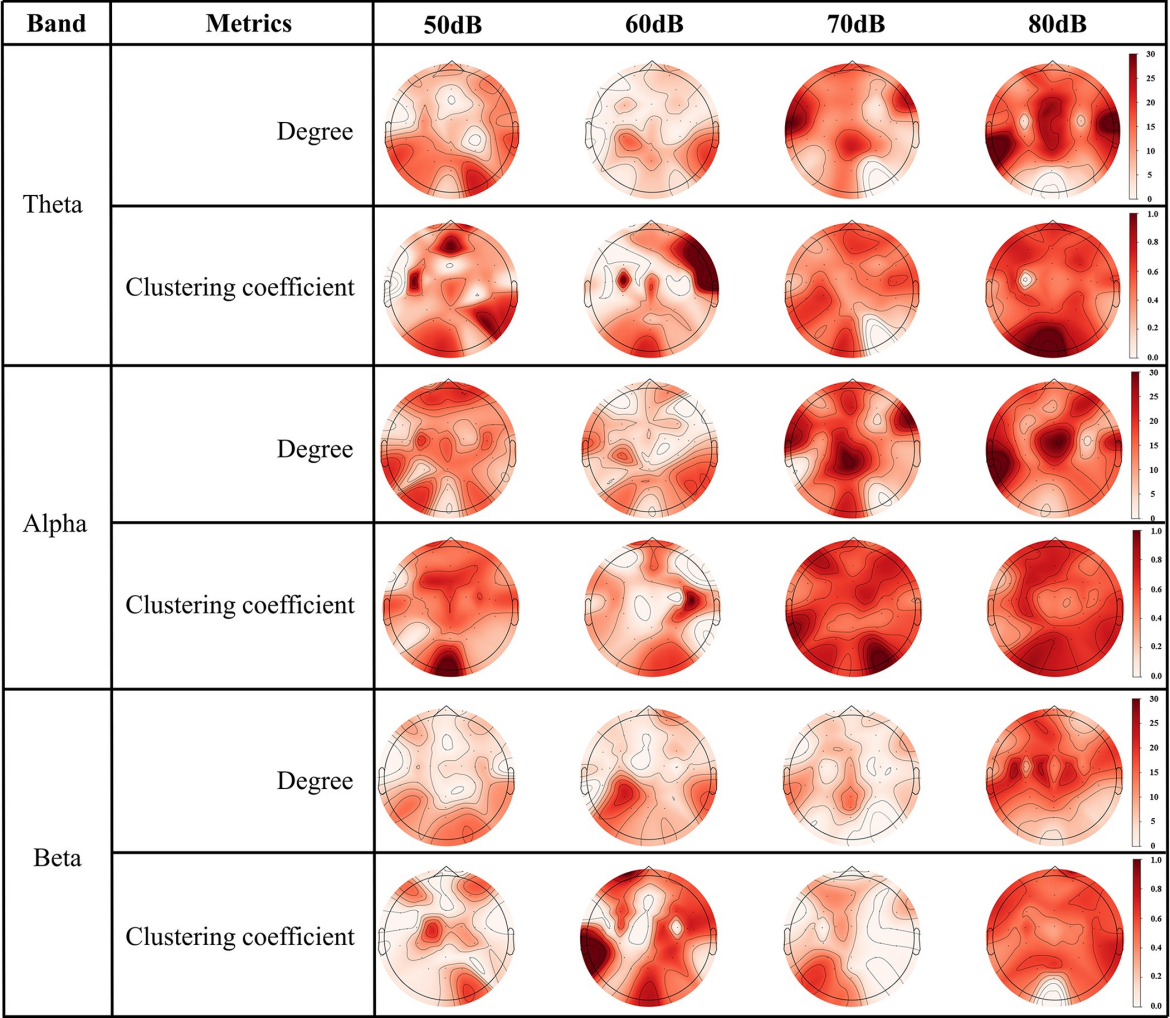
The local characterisation metrics of functional brain network at different noise levels. The results of the calculation of degree and clustering coefficients for 33 different electrodes in the theta, alpha and beta bands are shown in the figure.

The global efficiency describes the overall change of the network, and the use of local characterisation metrics (degree, clustering coefficient) allows us to understand the local characteristics of the network. Therefore, in this paper, the degree and clustering coefficients of 33 nodes are calculated and presented in the form of brain topography maps. Fig 8 shows the variation of the local characteristics for different noise levels. In the theta band, the degree (p < 0.0001) and the clustering coefficient (p = 0.0027) in the 60 dB noise environment were overall significantly lower than those in the 50 dB noise condition. The degree (p = 0.0284, p = 0.0417) and the clustering coefficient (p < 0.0001, p < 0.0001) in the 70 dB and 80 dB noise environments were overall significantly higher than those in the 50 dB noise condition. However, the degree (p = 0.3513) and the clustering coefficient (p = 0.562) were not significantly different between the 70 dB and 80 dB noise conditions. Meanwhile, the highest values of degree and clustering coefficient in the 70 dB and 80 dB high noise conditions were concentrated in the central and temporal lobes.

In the alpha band, the degree (p = 0.0004) and clustering coefficient (p = 0.0304) under 60 dB noise are overall significantly lower than that of the 50 dB noise condition. the degree (p = 0.0065, p < 0.0001) and clustering coefficients (p = 0.03411, p = 0.0002) under 70 dB and 80 dB noise are overall significantly higher than that of the 50 dB noise condition. In contrast to the theta band, the degree (p = 0.0119) and clustering coefficients (p = 0.0011) in the 80 dB noise condition also showed significantly higher values than in the 70 dB noise condition. The highest values of the degree in the 70 dB and 80 dB high noise conditions were similarly clustered in the central and temporal lobes. The highest values of the clustering coefficient were concentrated in the occipital lobe. This is consistent with theta band performance.

For the beta frequency band, only the degrees (p < 0.0001, p < 0.0001, p < 0.0001) below the 80 dB noise level show a significant increase with the 50 dB, 60 dB and 70 dB noise levels. The clustering coefficients at 80 dB noise level showed significant differences only with 50 dB and 70 dB noise levels (p < 0.0001, p < 0.0001).

### Reaction time gap

**Fig 9:**
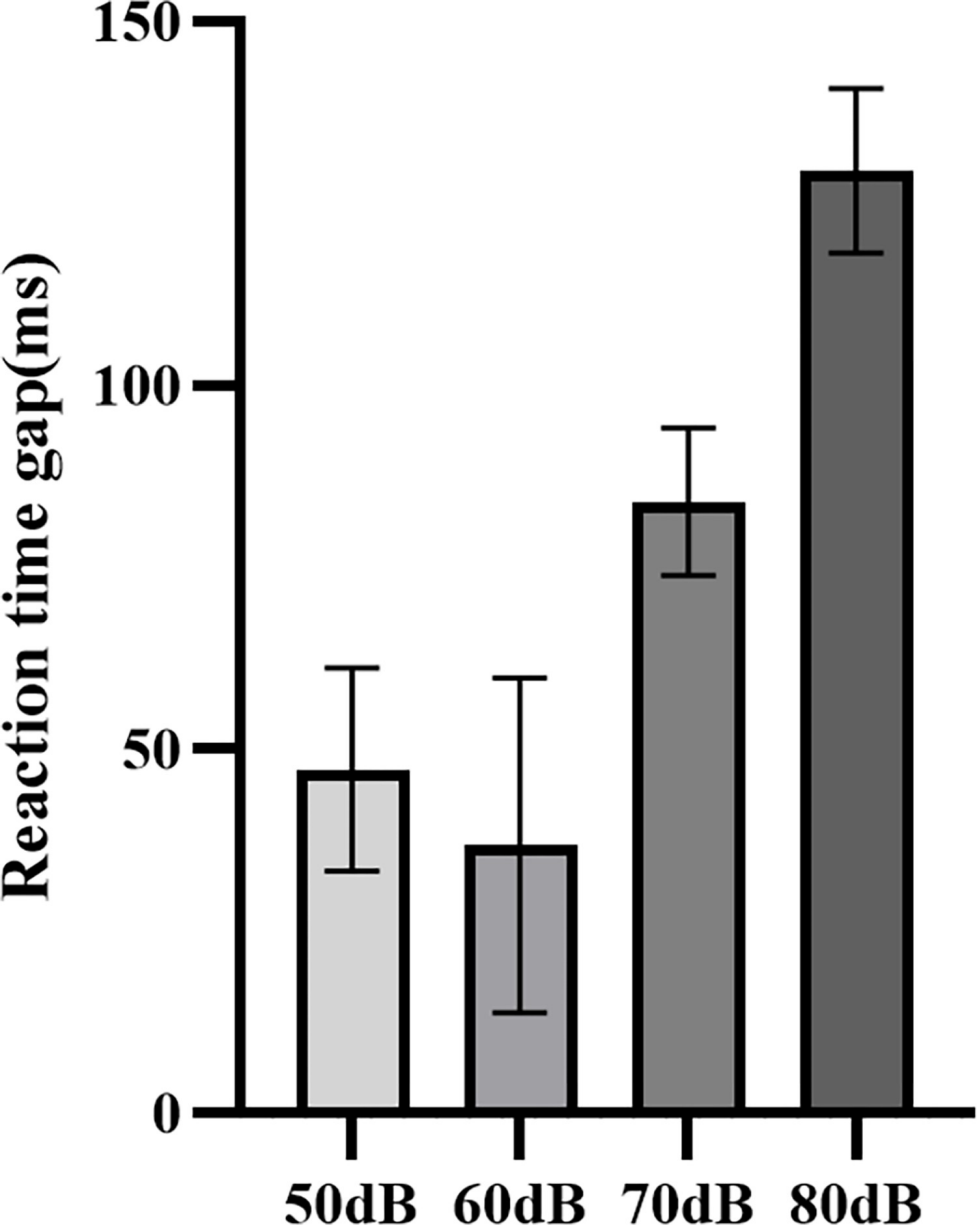
Difference in reaction time before and after driving in different noise levels.

The study utilised the difference in reaction time, specifically the increase in reaction time before and after driving, to characterise the subjects’ performance in the presence of different noises. As shown in Table 2, a one-way ANOVA was conducted to test the significance of the effect of noise on reaction time, revealing a significant impact of noise on reaction time (p = 0.0057<0.05). Fig 9 displays a comparison of reaction time differences under different noise conditions. Observation of Fig 9 shows that, consistent with the results of the functional brain network, the reaction time difference was significantly greater in the 70dB and 80dB noise conditions compared to 50dB and 60dB, and the reaction time was significantly longer. The study indicates that reaction time growth was more pronounced and driver behaviour was worse when exposed to high noise environments (70dB and 80dB) for the same driving task. However, the reaction time growth at 60 dB was the smallest compared to 50 dB.

**Table 2:** One-way ANOVA test table for reaction time gap in different noises.

	Sum of squares	df	MS	F	p
**Between-group variation**	24100	3	8033	6.978	**0.0057**
**Residual Error**	13815	12	1151		
**Total**	37916	15			

Bold text represents significant results (p < 0.05).

## Discussion

Traffic noise can cause adverse physiological effects on blood pressure and heart rate in extreme cases, and although short-term exposure to subway noise does not have serious effects on commuters [39], previous studies have demonstrated that noise increases the risk of driver fatigue behind the wheel and also reduces human decision-making performance [40]. The main objective of this study was to investigate the effects of train operating noise on the functional brain network regulation of drivers. To this end, this paper designed a simulated driving experiment under four noise levels (50, 60, 70 and 80 dB), constructed a functional brain network by recording EEG signals of the subjects, and analysed the brain network changes under different noise levels based on graph theory. It was found that in the theta and alpha frequency bands, different noise levels led to significant changes in the brain networks, and the functional brain networks of drivers in high-noise environments tended to self-regulate to make the networks more globally efficient. For the beta band, however, the significance of the results according to McNemar’s test and Wilcoxon signed-rank test was only found at the highest noise level (80 dB), suggesting that the beta band is not a good indicator of neural changes in drivers, which is in line with previous studies [12, 41].

The WPLI is a method for analysing the connectivity of functional brain networks. Its value reflects the degree of functional connectivity between different regions of the brain [42]. In this study, the WPLI results showed that for theta and alpha bands, the WPLI values at noise levels of 70 dB and 80 dB were significantly higher than those at 50 dB and 60 dB, indicating that high noise strengthens the connectivity of different brain regions and enhances cooperation between different regions of the brain. This phenomenon was evident in the functional brain network topology map, demonstrating that the brain networks exhibit considerably tighter connections and stronger connection strengths at 70 dB and 80 dB noise levels rather than at 50 dB and 60 dB. High noise levels have a detrimental effect on the driver’s attention [43, 44], necessitating the activation of further brain regions and strengthening of inter-region connections to achieve driving proficiency in high-noise environments. However, the noise levels ranged from 50dB to 80dB, and the WPLI value did not increase consistently with the increase in noise. At 60 dB, the connectivity of the functional brain network decreased compared to 50 dB. This suggests that appropriate noise can improve attention and maintain better performance [45]. This is also reflected in the degree of reaction time increase before and after driving.

During the graph-theoretic characterisation of functional brain networks, it was discovered that brain networks in 70 dB and 80 dB noise environments exhibited significantly greater global network efficiency, in comparison with 50 dB and 60 dB noise environments, in the theta and alpha frequency bands. This implies an improvement in the information transfer efficiency of the brain in high-noise environments. It should be noted that operating a train in a high-decibel atmosphere may result in drowsiness, thus impeding sustained attention. Consequently, the brain favours networks with elevated global efficiency [46]. In the local characterisation of degree and clustering coefficients, it was discovered that for both theta and alpha bands, test subjects exposed to 70dB and 80dB noise environments demonstrated increased degree and clustering coefficients at all nodes when compared to subjects in 50 dB and 60 dB noise environments. This suggests that all brain regions are activated [47]. Additionally, The temporal lobe is responsible for processing auditory information [48], and noise can increase the difficulty of processing auditory information. The central region controls human behavioural movements [49]. The occipital lobe processes visual information [50] and is responsible for perceiving vehicle motion. It is conceivable that this has resulted in the highest values of node degree and clustering coefficient occurring in the temporal, central and occipital lobes.

The statistical analysis indicates that the statistical significance between the theta frequency band at 70 dB and 80 dB is not significant. Within the theta and alpha frequency band, the functional connectivity of the brain network displayed a tendency to be tighter at 50 dB and the global efficiency of the brain network was higher at 50 dB compared to the 60 dB noise surroundings. The monotonous nature of the driving task, coupled with the quiet environment, may induce passive fatigue in drivers who have to resist fatigue to complete their tasks [51]. Accordingly, it is more cost-effective to keep train running noise levels below 70 dB. However, controlling the noise levels below 50 dB may increase the workload of drivers, necessitating further research into the physiological characteristics of drivers in low-noise environments.

## Conclusions

This study used a complex network approach to examine the functional brain network of drivers exposed to different noise levels in order to investigate the effects of train noise exposure on the driver’s brain network. The findings demonstrate that alterations in functional brain networks in the theta and alpha frequency bands are more indicative of the effects of noise. The high-noise environment (70 dB, 80 dB) can enhance synchrony between drivers’ brain regions and bring functional connectivity closer. Specifically, high-noise environments adjust functional brain network reorganisation in favour of brain networks with greater network global efficiency. Moreover, the higher degree and clustering coefficients of the temporal lobe, central region, and occipital lobe indicate that the high noise environment amplifies the challenge of processing audiovisual information. This study contributes to our understanding of how exposure to train running noise affects drivers’ neuromodulation. Additionally, from an applied perspective, this study emphasises the significance of implementing noise control measures to improve safety in public transportation.

Although this paper has achieved some results in analysing the functional brain networks of train drivers under different noise conditions. However, there are some limitations, including that the samples of this study were all students, and the lack of recruitment of train drivers with actual driving experience is a shortcoming of this study. In addition, the effect of the duration of noise exposure is also of interest for future research.

## Supporting information

S1 Dataset This data contains the WPLI, brain network feature metrics, and behavioural metrics. (ZIP)
